# Food Sources of Energy and Macronutrient Intakes among Infants from 6 to 12 Months of Age: The Growing Up in Singapore Towards Healthy Outcomes (GUSTO) Study

**DOI:** 10.3390/ijerph15030488

**Published:** 2018-03-10

**Authors:** Shan-Xuan Lim, Jia-Ying Toh, Linde van Lee, Wee-Meng Han, Lynette Pei-Chi Shek, Kok-Hian Tan, Fabian Yap, Keith M. Godfrey, Yap-Seng Chong, Mary Foong-Fong Chong

**Affiliations:** 1 Singapore Institute for Clinical Sciences, Agency for Science, Technology and Research (A*STAR), Singapore 117609, Singapore; shan_xuan_lim@u.nus.edu (S.-X.L.); toh_jia_ying@sics.a-star.edu.sg (J.-Y.T.); lindevanlee@hotmail.com (L.v.L.); lynette_shek@nuhs.edu.sg (L.P.-C.S.); yap_seng_chong@nuhs.edu.sg (Y.-S.C.); 2 Food Science and Technology Programme, Department of Chemistry, National University of Singapore, Singapore 117543, Singapore; 3 Department of Nutrition and Dietetics, KK Women’s and Children’s Hospital, 100 Bukit Timah Road, Singapore 229899, Singapore; han.wee.meng@kkh.com.sg; 4 Department of Paediatrics, Yong Loo Lin School of Medicine, National University of Singapore, 1E Kent Ridge Road, Singapore 119228, Singapore; 5 Khoo Teck Puat-National University Children’s Medical Institute, National University Hospital, 5 Lower Kent Ridge Road, Singapore 119074, Singapore; 6 Department of Maternal Fetal Medicine, KK Women’s and Children’s Hospital, Singapore 229899, Singapore; tan.kok.hian@singhealth.com.sg; 7 Duke-NUS Medical School, Singapore 169857, Singapore; fabian.yap.kp@kkh.com.sg; 8 Department of Paediatrics, KK Women’s and Children’s Hospital, 100 Bukit Timah Road, Singapore 229899, Singapore; 9 Lee Kong Chian School of Medicine, Nanyang Technological University, Experimental Medicine Building, Nanyang Drive, Singapore 636921, Singapore; 10 MRC Lifecourse Epidemiology Unit & NIHR Southampton Biomedical Research Centre, University of Southampton & University Hospital Southampton NHS Foundation Trust, Southampton SO16 6YD, UK; kmg@mrc.soton.ac.uk; 11 Department of Obstetrics and Gynaecology, Yong Loo Lin School of Medicine, National University of Singapore, 1E Kent Ridge Road, Singapore 119228, Singapore; 12 Saw Swee Hock School of Public Health, National University of Singapore, 12 Science Drive 2, Singapore 117549, Singapore; 13 Clinical Nutrition Research Centre, Singapore Institute for Clinical Sciences, Agency for Science, Technology and Research (A*STAR), Centre for Translational Medicine, Medical Drive #07-02, MD 6 Building, Yong Loo Lin School of Medicine, Singapore 117599, Singapore

**Keywords:** infant diet, complementary feeding, weaning, food sources, energy, macronutrients, Asian

## Abstract

Adequate nutrition during complementary feeding is important for the growth, development and well-being of children. We aim to examine the energy and macronutrient intake composition and their main food sources in a mother–offspring cohort study in Singapore. The diets of infants were assessed by 24 h dietary recalls or food diaries collected from mothers when their offspring were 6 (n = 760), 9 (n = 893) and 12 (n = 907) months of age. Food sources of energy and macronutrients were determined using the population proportion methodology. Energy intakes per day (kcal; mean (standard deviation, SD)) of these infants were 640 (158) at 6 months, 675 (173) at 9 months, and 761 (208) at 12 months. Infant formula, breastmilk and infant cereals were the top three food sources of energy and macronutrient intakes in infants through the period 6 to 12 months. Other main energy and carbohydrate sources at 9 and 12 months of age were rice porridge, infant biscuits and fresh fruits, while fish, red meat and eggs were the other main protein and total fat sources. Breast-fed and mixed-fed infants had a more varied diet as compared to formula-fed infants. Formula-fed infants had consistently higher protein and lower total fat consumption compared to those who were breastfed. An understanding of these main food sources during complementary feeding can inform local dietary recommendations and policies.

## 1. Introduction

Complementary feeding or weaning is the process whereby solid foods and other liquids, in addition to milk, are introduced to meet a growing infant’s nutritional requirements [1]. Macronutrient composition of the early diet are primary factors influencing childhood growth patterns and the subsequent risk of non-communicable diseases [2]. An understanding of the food sources of infants’ energy and macronutrient intakes is important to enable targeted interventions to improve infants’ nutritional needs and inform local dietary recommendations.

There has been a growing interest in examining infants’ nutrient intakes and their food sources in several countries worldwide. The Feeding Infants and Toddlers Study (FITS) in the US and the Maternal Infant Nutrition Growth (MING) Study in China have analysed food sources of nutrients in 6- to 11-month-old infants and reported that infant formula and breastmilk were key contributors to energy intakes [3,4]. The prevalence of macronutrient inadequacy was reported to be low in 6 to 11-month-old FITS infants, while infants belonging to the MING study were reported to have total fat consumption below recommendations, in part due to a reduction in milk intakes as table foods were introduced [4]. Two other studies in Australia have reported that 3% of infants at 6 months and 58.2% at 9 months of age were consuming energy-dense snacks or sweet foods and beverages (e.g., fast food, lollipops, soft drinks) [5,6]. In infants and children (6 months to 12 years of age) from four South-east Asian countries, stark differences in energy and macronutrient intakes were observed between those from urban and rural areas [7,8,9,10]. These differences reflect the phenomenon of ‘double burden of malnutrition’, where undernutrition and overnutrition coexist [7,8,9,10]. Taken together, these studies highlight that infants from different populations have different and various nutritional concerns that need to be addressed.

In Singapore, the local Health Promotion Board recommends exclusive breastfeeding for at least 6 months, followed by the introduction of solid foods such as foods rich in iron (e.g., iron-fortified cereals), vegetables and fruits (which enhance iron absorption), to supplement breastmilk or formula [11]. However, there is a lack of data on the dietary intakes and food sources of infants aged 6 to 12 months and the nutritional status of our infants is not known. In this study, we sought to examine the food sources and macronutrient intakes among multi-ethnic Asian infants using data from the Growing Up in Singapore Towards healthy Outcomes (GUSTO) mother-offspring cohort study. We aim to identify the main food sources of energy and macronutrient intakes of these infants and examine differences in food sources among infants who were formula-fed (FF; given formula), breast-fed (BF; given breastmilk) and mixed-fed (MF; given both breastmilk and formula).

## 2. Materials and Methods

### 2.1. Study Population

Data was acquired from the GUSTO study. In summary, a total of 1247 pregnant women, aged 18 and above, were recruited between June 2009 and September 2010 from local hospitals during their antenatal clinic visits. The inclusion criteria were that they were Singapore citizens or permanent residents having the intentions to reside locally for the next 5 years and delivering at either of the maternity units of the two local hospitals. Mothers also had to be of a homogenous parental Chinese, Malay or Indian ethnic background and willing to donate cord, cord blood and placenta after delivery. Women with type I diabetes mellitus or receiving any chemotherapy or psychotropic drugs were excluded. Written informed consent was obtained from all participants prior to the study. Further details of the recruitment process, cohort profile and data collection procedures of the GUSTO study have been published [12]. The study was approved by the National Healthcare Group Domain Specific Review Board (reference number D/09/021) and the SingHealth Centralized Institutional Review Board (reference number 2009/280/D).

### 2.2. Maternal and Infant Characteristics

Maternal socioeconomic characteristics such as ethnicity, education level and marital status were collected from participants during the recruitment phase at 12 weeks of gestation. Data on smoking or alcohol consumption status and pregnancy body mass index (BMI) at 26 weeks of gestation were obtained during participants’ clinic visits between 26 to 28 weeks of gestation. Other characteristics such as infant gender, gestational age and parity were determined from birth records.

### 2.3. Infant Dietary Assessment

Prior to the postnatal clinic visits at 6, 9 and 12 months, mothers were mailed a 3-day food diary to record their infant’s diet. A 24 h recall was administered by trained personnel to mothers who did not return the food diaries during postnatal clinic visits [13]. In order to have a 1-day dietary record for every infant, a 1-day record was randomly chosen from the 3-day food diaries (some of which were completed for 1 or 2 days only). This is to allow for comparability with one 24 h recall conducted on mothers who did not return completed food diaries. Data from either the 24 h dietary recalls (n at 6, 9, 12 months: 483, 600, 506, respectively) or a 1-day record from food diaries (n at 6, 9, 12 months: 277, 293, 401, respectively) were used for dietary analyses to increase the sample size. A moderately strong correlation (overall mean r = 0.647) of the 1-day record with the two other days using a subset of infants with complete 3-day food diaries (n = 163) has been previously established [14].

Based on the type of milk consumed as recorded in the dietary records, infants were then assigned into one of the three milk-feed types: breast-fed, formula-fed and mixed fed at ages 6, 9 and 12 months. Almost all of the infants, regardless of milk-feed type, were given complementary foods. BF infants who were directly breastfed were assumed to consume total volumes of 780 mL (6 months) and 600 mL (9 and 12 months) per day as was done by a previous study [15]. For mix-fed infants, the volume of unmeasured breastmilk consumed was obtained by subtracting formula intake from these totals. Expressed breastmilk volumes were quantified based on actual volumes recorded. Nutrients derived from breastmilk at the respective age groups were estimated based on existing literature [16]. Nutrient analysis of foods consumed was performed using the nutrient analysis software, Dietplan 7 (Forest Field Software). The local food-composition database was mainly used for the analysis of dietary records of this study, with modifications made for inaccuracies found [17]. For mixed dishes unavailable in the local database, nutrient analyses of recipes were performed using the nutrient software. Nutrient information from food labels (especially of infant products) or the United States Department of Agriculture (USDA) national nutrient database was obtained for any other food items not found in the local database [18].

Each food item was then assigned to one of the 72 sub-food groups within 18 food groups based on type of food or similarities in nutrient content, which is conceptually similar to previous studies [3,4] and was used to categorize the foods consumed locally (online Supplementary Table S1). In this classification scheme, the cooking methods of several food items were specified as either boiled and steamed (healthier cooking methods; uses little or no oil) or deep-fried (less healthy cooking method; uses large amounts of oil).

Composite dishes, with the exception of infant products, were broken down into single food items for categorization. The contribution of each food group to the total intake of energy and macronutrient was calculated stratified by milk-feed group and time point using the population proportion formula as follows [19]. This method calculates the food sources contributing to energy and macronutrient on the group level. In order to calculate the percentage contribution of each food group to energy, we first summed up the energy values of all food items under the same food group, which forms the numerator of the formula. Next, the denominator of the formula was calculated by adding up the energy values contributed by all food items from all food groups. The same method was subsequently applied to calculate the percentage contribution of each food group to each of the macronutrient. Only the food contributions to energy and macronutrient intakes of at least 1% were presented.

Percentage contribution of each food group (%)

$$\frac{\mathrm{Sum}\;\mathrm{of}\;a\;\mathrm{given}\;\mathrm{energy}\;\mathrm{or}\;\mathrm{macronutrient}\;\mathrm{for}\;\mathrm{all}\;\mathrm{individuals}\;\mathrm{for}\;\mathrm{each}\;\mathrm{food}\;\mathrm{group}}{\mathrm{Total}\;\mathrm{energy}\;\mathrm{or}\;\mathrm{macronutrient}\;\mathrm{consumed}\;\mathrm{by}\;\mathrm{all}\;\mathrm{individuals}\;\mathrm{for}\;\mathrm{all}\;\mathrm{food}\;\mathrm{groups}\;}\;\times \;100$$

Macronutrient intakes were expressed in terms of percentage contribution to total energy for each infant. Protein and total fat were taken to yield 4 and 9 kcal of energy per gram respectively. Carbohydrate intake was then calculated by subtracting the % protein and % total fat from 100%.

### 2.4. Statistical Analysis

Group mean and standard deviation were then calculated for energy and energy-adjusted macronutrients (% of energy) for BF, FF and MF infants. A one-way analysis of variance (ANOVA) test followed by post-hoc analysis with Bonferroni-corrected *p*-values were used to assess differences in energy and macronutrient intakes among milk-feed groups. Due to the differing energy requirements between gender, we have also calculated group mean and standard deviation for each gender for all infants and infants by milk-feed type.

A comparison of baseline maternal and infant characteristics including socio-economic factors of included and excluded subjects for the cross sectional analyses was performed using Pearson’s chi-squared tests. All statistical analyses were performed using IBM SPSS Statistics Version 20.0 (IBM Corp., Armonk, NY, USA). Statistical significance was identified by 2-tailed *p*-values of less than 0.05.

## 3. Results

A total of 1176 infants were delivered from the original cohort of 1247 pregnant women recruited [12]. Of these live births, 1051 infants, who had available dietary data for at least one of the 3 time points (6 months (n = 760), 9 months (n = 893) and 12 months of age (n = 907)), were included for this study (online Supplementary Figure S1).

### 3.1. Characteristics of Participants

The maternal and infant characteristics of the study population are tabulated in Table 1. In this sample of included infants, there was a larger proportion of born to term (37 weeks) male infants, who were not the first child and were breast-fed for more than three months. A larger proportion of these infants were also born to mothers who were younger, were Chinese, had higher education and household income, were employed, had lower BMI at 26 weeks of pregnancy and who did not consume alcohol or smoke before and during pregnancy.

A comparison of the maternal and infant characteristics showed that included subjects tended have a full-term (37 weeks) infant, breast-fed for more than 3 months, were older, of a Chinese ethnicity, more highly educated, had higher household income and were more likely to be non-smokers before pregnancy as compared to excluded subjects (Table 1).

### 3.2. Energy and Macronutrient Intakes of Infants by Milk-Feed Type

The majority of the cohort was fed with formula milk (without breastmilk) at 6, 9 and 12 months of age (63.2%, 72.8%, and 79.9%, respectively). The prevalence of infants fed with breastmilk (without formula) in the entire cohort, at the respective time points, was low from 6 to 12 months of age (online Supplementary Figure S2).

The mean energy intakes of all infants at 6, 9 and 12 months were 640, 675 and 761 kcal, respectively (Table 2). The only significant difference in mean energy intakes, among milk feed types, was observed at 9 months of age (*p* < 0.001) (Table 2). FF and MF infants had higher energy intakes as compared to BF infants. Overall, the local estimated energy requirements for both male and female infants at 6 (M: 600 kcal; F: 560 kcal), 9 (M: 670 kcal; F: 620 kcal) and 12 (M: 740 kcal; F: 640 kcal) months were met, with the exception of 9-month-old breast fed infants and 12-month-old breast-fed male infants (Table 3) [20].

Increasing intakes from 6 to 12 months of age were observed for protein (9.9 to 14.5 TE% (total energy %)), carbohydrate (48.1 to 50.9 TE%) and dietary fibre (2.5 to 4.4 g per 1000 kcal TE). Total fat intake, however, decreased from 42.0 TE% at 6 months to 34.6 TE% at 12 months (Table 2). For the macronutrient levels at 12 months, those recommended by the Institute of Medicine (IOM) i.e., protein (5–20 TE%), total fat (30–40 TE%) and carbohydrate (45–65 TE%) were all met (Table 2) [21].

Macronutrient intakes (TE%) differed significantly across the various milk-feed groups at 6, 9 and 12 months of age (Table 2). FF infants followed by MF infants consistently had the highest protein (TE%) intakes as compared to BF infants at 6, 9 and 12 months of age, respectively. An overall reverse trend was observed for total fat intakes (TE%) at 6, 9 and 12 months of age: BF infants followed by MF infants had the highest intakes as compared to FF infants. However, at 9 months of age, MF infants had a slightly higher total fat intake compared to BF infants. The trend for carbohydrate intakes at 6 and 12 months is similar to that of protein intakes, with FF infants having the highest intakes and BF the lowest.

### 3.3. Main Food Sources of Energy and Macronutrient of Infants

**Figure 1:**
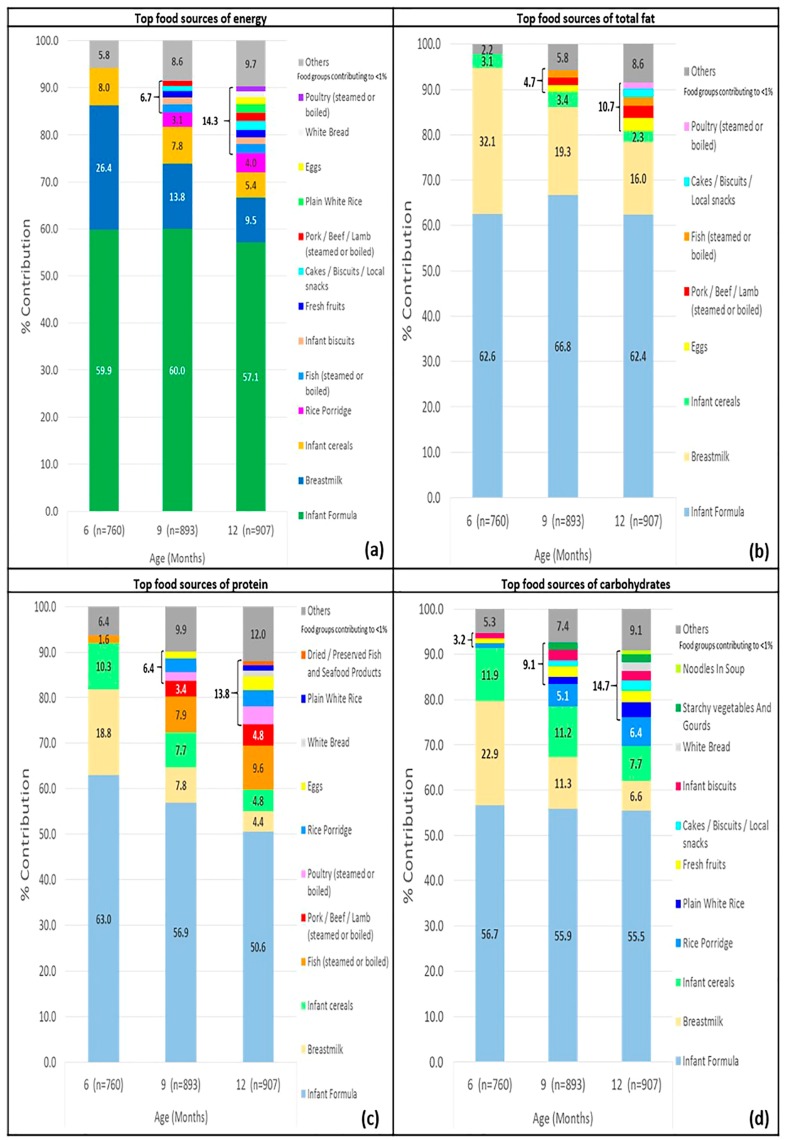
Cross-sectional analyses of top food sources of (**a**) energy; (**b**) total fat; (**c**) protein and (**d**) carbohydrate of Growing Up in Singapore Towards healthy Outcomes (GUSTO) infants at 6, 9 and 12 months of age.

The main food sources of energy and each of the macronutrient at 6, 9 and 12 months of age are shown in Figure 1 and further details can be found in online Supplementary Table S2. In general, infants consumed an increasingly varied diet from 6 to 12 months of age, evident by the increase in number of food sources from 6 to 12 months: 3 to 13 food sources for energy, 3 to 8 food sources for total fat, 4 to 11 food sources for protein and 6 to 11 food sources for carbohydrate. Infant formula, breastmilk and infant cereals were consistently ranked as the top three food sources of energy throughout the period 6 to 12 months.

The total contribution of the top three food sources (infant formula, breastmilk and infant cereals) to energy intakes were 94.3%, 81.6% and 72.0%, at 6, 9 and 12 months, respectively. The three same foods are also the major contributors of total fat, protein and carbohydrate intakes at the three time points (Figure 1a–d). Rice porridge was the next key source of energy after the top three sources aforementioned, contributing to 3.1% and 4.0% of total energy at 9 and 12 months, respectively (Figure 1a). For 9-month-old infants, the other key food sources contributing to a total of 6.7% of energy included fish, red meat, infant biscuits, snacks (cakes, biscuits, local snacks) and fresh fruits (fruits that were mashed or cooked to achieve the correct texture, excluding fresh or commercial fruit juices). For 12-month-old infants, the other key food sources included eggs, poultry, red meat, white rice and white bread, contributing in total to 14.3% of energy.

A relatively small number of other food sources contributed to infants’ total fat intakes (Figure 1b). Eggs, red meat and fish provided a total of 4.7% and 7.3% total fat at 9 and 12 months, respectively. At 12 months, snack items (cakes, biscuits, local snacks) and poultry made up 3.2% of total fat intakes. Cooking oil and deep-fried food items accounted for <1% of energy and macronutrient intakes, reflecting the common cooking methods of steaming and boiling in these age groups.

For protein intakes, fish was ranked next after the top three food sources at 6, 9 and 12 months respectively (1.6%, 7.9% and 9.6% of total protein respectively) (Figure 1c). Red meat and poultry emerged next (contributing 5.3% and 8.7% protein at 9 and 12 months, respectively), followed by rice porridge (3.0% and 3.5% total protein respectively) (online Supplementary Table S2).

For total carbohydrate intakes, rice porridge was ranked next after the top three food sources at 9 and 12 months of age (5.1% and 6.4% total carbohydrate respectively) (Figure 1d). At these two time points, fresh fruits and starchy vegetables/gourds totalled up to 3.8% and 4.3% of total carbohydrate in infants. At 6 months of age, the other foods introduced were infant biscuits, fresh fruits and rice porridge (online Supplementary Table S2).

For ease of interpretation, dietary fibre intakes were classified using the main food groups (online Supplementary Figure S3). The top three food sources of dietary fibre were, in descending order: infant products, vegetables, fruits and juices (inclusive of fresh fruits, fruit juices and dried fruits) at 6, 9 and 12 months of age (contributing 87.1%, 70.9% and 58.7% dietary fibre at the respective time points). Other sources of dietary fibre, at 6, 9 and 12 months, were rice and grain alternatives and legumes and pulses, which together contributed to 7.1%, 17.0% and 24.3% dietary fibre, respectively.

### 3.4. Key Food Sources of Energy and Macronutrient of Infants by Milk-Feed Type

**Figure 2:**
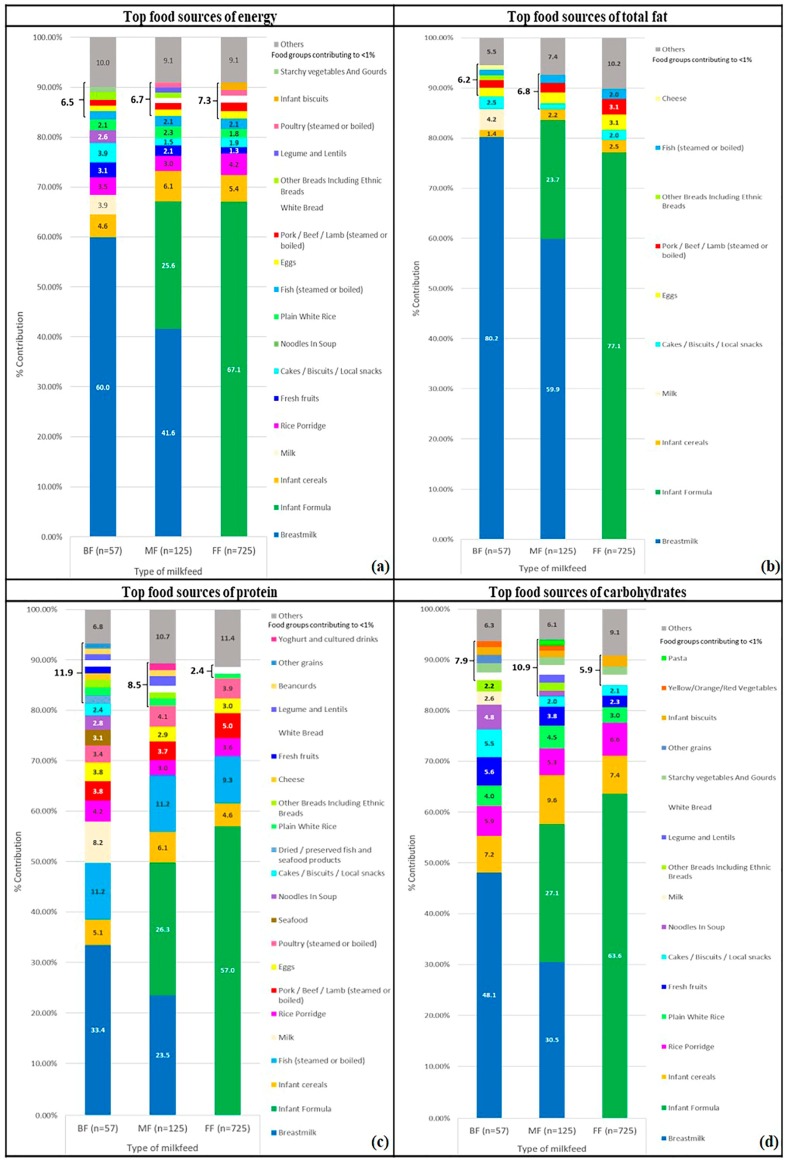
Cross-sectional analyses of top food sources of (**a**) energy; (**b**) total fat; (**c**) protein and (**d**) carbohydrate of BF, MF and FF Growing Up in Singapore Towards healthy Outcomes (GUSTO) infants at 12 months of age.

While the variety of weaning foods among BF, FF and MF infants were largely similar at 6 months, BF and MF infants had slightly more varied diets as compared to FF infants at 9 and 12 months of age (online Supplementary Tables S3–S5 and Figure 2). At 6 months, the only non-infant specific source of carbohydrate in BF and MF infants was fresh fruits, while it was rice porridge for the FF infants’ diets. For key dietary fibre sources, infant formula was the main contributor to MF and FF infants’ diets, while fresh fruits emerged as the top dietary fibre source in BF infants’ intakes at 6 months (online Supplementary Table S3). Patterns observed for key food sources of energy and macronutrients were fairly similar between 9 and 12 months of age, other than the increased number of food sources at 12 months. For instance, there were 9, 8 and 7 key food sources contributing to 9-month-old BF, MF and FF infants’ energy intakes respectively (online Supplementary Table S4). At 12 months, these numbers increased to 13, 14 and 12 for BF, MF and FF infants respectively (online Supplementary Table S5 and Figure 2). Subsequent paragraphs are focused on the comparison of key food sources among BF, MF and FF infants at 12 months of age.

In terms of energy intakes, fresh fruits contributed to 3.1% and 2.1% energy of 12-month old BF and MF infants’ diets, respectively, but only 1.3% energy for FF infants at 12 months. BF infants, at 12 months old, had at least 1% of energy contributed from starchy vegetables and gourds. This was, however, not observed in MF and FF infants. For MF and FF infants, rice porridge remains to be a major contributor to energy intakes, at 12 months of age, amongst the non-infant food products.

Milk (from cow or goat sources) and cheese contributed to food sources of total fat in 12-month-old BF infants’ diet, but less than 1% in that of the 12-month-old FF and MF infants’ diets. Plant sources of legumes/lentils and bean curds made up at least 2% total protein of 12-month-old BF and MF infants’ diets, while FF infants consume less than 1% from these sources.

## 4. Discussion

In this study, we presented the main food sources of energy and macronutrient intakes of 6-, 9- and 12-month-old GUSTO infants. Our results showed that infant formula, breastmilk and infant cereals were the top three food sources of energy through the period 6 to 12 months. Breast-fed and mixed-fed infants had a more varied diet as compared to formula-fed infants. From 6 to 12 months of age, the majority of the GUSTO infants were formula-fed and had a higher protein and lower total fat consumption than the infants who had been breast-fed.

### 4.1. Energy and Macronutrient Intakes of All Infants and by Milk-Feed Type

The prevalence of breast-fed infants in our study was low at 6 and 12 months (16% and 6% of infants, respectively). The general trend of macronutrient intakes (increasing protein and carbohydrate, decreasing total fat) in GUSTO infants as well as the range of intakes (TE%) from 6 to 12 months of age was similar to observations in FITS infants [22]. The decrease in total fat intakes in GUSTO infants was mostly due to the decreasing consumption of breastmilk by all infants (32.1 to 16.0% of total fat at 6 and 12 months, respectively). The increase in protein and carbohydrate intakes in GUSTO infants, however, was likely to be due to the introduction of complementary foods such as red meat and rice, respectively. According to the World Health Organisation (WHO), for infants/toddlers between weaning and under 2 years of age, 30 to 40% of energy from total fat is recommended for immune defence and neural development [23]. In our study, mean intakes from total fat ranged from 34.6 to 42.0 TE%, which were similar to WHO recommendations.

Akin to the Dortmund Nutritional and Anthropometric Longitudinal Designed (DONALD) study, FF infants in GUSTO consistently had higher carbohydrate and protein intakes, but lower total fat intake as compared to the BF infants [24]. From 6 to 12 months of age, the majority of the GUSTO infants, who were formula-fed, had a higher protein and lower total fat consumption. This is in line with findings on 9-month-old Danish and Australian infants who had consumed breastmilk having higher total fat and lower protein intakes as compared to those who consumed only formula milk [25,26].

Comparing the average energy intakes of BF infants to the local recommendations, we observed that the average energy intake of 9-month-old BF infants, both males and females, falls short of the estimated average energy requirement (EAR) of 670 kcal and 620 kcal, respectively. Additionally, at 9 months, less than half of the BF infants met the EAR (18.4% of male BF infants and 33.3% of female BF infants met the EAR). This explains the significant difference in mean energy intakes among milk-feed types observed at 9 months of age. At 12 months, male BF infants’ mean energy intake was 720 kcal, which was lower than their MF and FF counterparts, and also below the EAR of 12-month-old male infants (740 kcal). This suggests a possibility that breast-fed infants at the ages of 9 and 12 months may not be receiving adequate energy from complementary foods, although we recognize that the limited sample size of BF infants in our study does not allow us to be conclusive on this. Addtionally, the local EARs are applicable to the general population of infants and not specifically to BF infants.

### 4.2. Top Food Sources of Energy and Macronutrient of Infants

In our study, where 6 to 12 months old infants were followed prospectively, the top three food sources of energy (infant formula, breastmilk and infant cereals) concurs with those previously reported in 6- to 11-month-old FITS infants [3,22].

Food sources of energy and macronutrient of GUSTO infants shared several similarities to infants from the FITS [3], MING [4], National Health and Nutrition Examination Survey (NHANES) [27] and Cambridge [28] studies. Rice was the top table food contributor to energy intakes of both GUSTO and MING infants, although a lower contribution of rice porridge in 9- and 12-month-old GUSTO infants (3.1 and 4.0 TE%, respectively) was observed as compared to 6- to 11-month-old MING infants (10.0% from rice) [4]. Rice and other flour-based products such as bread similarly made up a large proportion of the key food sources of carbohydrate in GUSTO, FITS and MING infants’ diets [3,4]. Protein from animal sources, such as poultry and red meat, contributed substantially to infants’ intakes in all three studies [3,4]. While breastmilk was a common contributor of total fat intakes, GUSTO infants (mean of 22.5% across 3 time points) had the lowest contribution compared to Cambridge (38.0%), FITS (39.5%) and MING (28.0%) infants [3,4,28], likely due to GUSTO infants being predominantly formula-fed. Eggs were consistently observed to be a common food source of total fat in GUSTO, NHANES and Cambridge infants [27,28]. Another similarity was the large percentage of dietary fibre intakes contributed from infant products, followed by various vegetables and fruits in the diets of GUSTO, FITS and MING infants [3,4].

However, differences in key food sources of energy and macronutrient amongst GUSTO infants and their 6- to 11-month-old FITS and MING counterparts were also noted. Whilst the top three food sources of energy and macronutrient of 6- to 12-month-old GUSTO infants were consistently infant-specific sources (infant formula, breastmilk and infant cereals), other table foods/beverages contributed to FITS and MING infants’ top three energy and macronutrient sources. Unlike the GUSTO and FITS infants who had infant cereals as the third key food source of energy, this was rice in the MING infants [4]. Correspondingly, rice was ranked as the second highest source of carbohydrate in MING infants’ diets (16%), whilst rice and rice porridge collectively contributed to less than 10% carbohydrate in GUSTO infants’ diets and less than 1% in FITS infants’ diets [3,4]. It was noteworthy that pure fruit juice, contributed a significant 7.8% to FITS infants’ diets [3] (third highest source of carbohydrate), but was not a main feature in the diets of both GUSTO and MING infants, who consumed at least 1% of carbohydrate from fresh fruits [4]. Table foods, such as non-formula milk (e.g., cow/goat/soy milk) and eggs were identified as one of the top three total fat and protein sources in the FITS and MING infants, respectively [3,4]. This is unlike the GUSTO infants, who had infant-specific food sources contributing to a large proportion of their total fat and protein intakes. Taken together, it appears that for the same age group, FITS and MING infants, as compared to GUSTO infants, have thus begun to consume significantly larger proportions of table foods/beverages. These differences also highlight that GUSTO infants obtain their macronutrient needs from a smaller number of food groups than their counterparts from the FITS and MING studies. Consuming a wide variety of food in early life would enable nutrient requirements to be satisfied and sets the foundation for having a balanced diet later in life [3].

A comparison of the number of key food sources of macronutrient of 12-month-old GUSTO infants showed slightly fewer total fat food sources as compared to those of protein and carbohydrate (8, 11, and 11 for total fat, protein and carbohydrate, respectively). Such a trend was similarly observed in 6- to 11-month-old FITS and MING infants [3,4]. This is likely due to a predominant milk-based diet in infants at this stage and that both infant formula and breastmilk are relatively rich sources of total fat. The contributions of other table foods to total fat, unlike food sources of protein and carbohydrate, were thus less prominent.

Our results, on key food sources by milk-feed type concur with a previous study on Mexican infants [29], where infants who consumed breastmilk (BF or MF infants) had a slightly more varied diet in terms of the number of food sources of energy as compared to FF infants. Looking at the specific food sources, another study showed that vegetables made a higher contribution to 9-month-old breast-fed Danish infants’ diets as compared their non-breast fed counterparts [25]. Similarly, GUSTO infants, where BF and MF infants had fresh fruits contributing to at least 1% of carbohydrate intakes, consumed more vegetables as well as plant-based protein in general than FF infants. Dietary fibre intakes of BF and MF infants were mainly contributed by fresh fruits and legumes/lentils at 12 months, while FF infants remained reliant on infant formula for dietary fibre at 6, 9 and 12 months of age.

### 4.3. Strengths and Limitations

This study presents a detailed description of the macronutrient intakes and their food sources in weaning diets from 6 to 12 months of age in Singapore. In addition, infants sampled in this study were followed prospectively through the period of 6 to 12 months of age. This would enable the close tracking of infants’ diets and could reduce the between-subject variation in food sources, observed in other similar studies which sampled different infants at a few time points concurrently.

The first limitation of our study is the use of a self-reported, single-day dietary record or 24 h recall of infants’ diets at 6, 9 and 12 months of age, which may not be representative of their usual intake during the weaning period. In addition, our dietary data was obtained from a mixture of 24h recall and one day of the three-day food diary. However, a moderately strong correlation of a single-day dietary intake with dietary intakes from two other days within the GUSTO cohort has been previously established [14], suggesting small variations in day to day food group intakes of infants at this age group.

Secondly, we acknowledge that the sample used in our study is not representative of the 6- to 12-month-old infants in Singapore as mothers were recruited from only 2 major public maternity hospitals. However, this is the only study, to date, that has been carried out to characterize the complementary foods of local infants. Additionally, based on official records, public hospitals (47.2%) account for slightly less deliveries as compared to private ones (52.8%) [30]. Further studies examining the dietary habits of a representative local population at infancy may be warranted to determine if similar findings can be obtained.

Thirdly, it was observed that the number of breast-fed infants at 12 months (n = 57) was relatively low as compared to the other milk-feed types at the same time point. Thus, the results may not be generalizable to all breast-fed infants.

Fourthly, the estimation of intakes and macronutrient composition of breastmilk were based on available literature and may not represent actual intakes. Hence, there may be slight deviations in the actual nutrient intakes of infants who consumed breastmilk. Similar to other studies [3,4,25], this was the next best alternative when laboratory analysis of breastmilk was not feasible in the study.

Lastly, as women and their infants with food allergies or intolerances were included in this study, their macronutrient intakes and food sources may have differed from the rest, due to an avoidance of certain foods. However, this should have a minimal impact on our findings as only a low food-allergy prevalence (2.9% at 12 months) was observed in the GUSTO cohort [31].

### 4.4. Implications for Local Recommendations and Policies

From our study, we observed that energy and macronutrient intakes of GUSTO infants at 6, 9 and 12 months of age have generally been met, except for BF infants who were consuming less than their energy requirements from 9 to 12 months of age. Additionally, in line with recommendations to introduce iron-rich complementary foods to supplement breastmilk or formula from 6 to 12 months of age [11], infant cereal was observed to be one of the top three food sources of energy and macronutrient. Vegetables, fruits and meat/fish were also increasingly introduced as the infants grew older, reflecting an increasing variety of complementary foods with age.

Our study raised several concerns, which could be further addressed in local recommendations. Firstly, FF infants, especially at 12 months old, were consuming a smaller variety of foods compared to MF and BF infants. We postulate that this could be due to a misconception that infant formula would provide all essential nutrients and hence exposure to a greater variety of foods is not as necessary to provide all the required nutrients. Secondly, “cakes, biscuits and local snacks”, a source of discretionary calories, was observed to contribute to a higher percentage of energy to BF infants’ diets as compared to their MF and FF counterparts, at 12 months of age. It is not clear why this is so, but it highlights an important point of educating mothers about the use of energy-dense, yet nutrient-poor snacks as part of the current recommendations.

Thirdly, while the benefits of breastfeeding to maternal and child health are well established [32], a relatively low rate of breastfeeding was still observed within the GUSTO cohort when compared with other countries. More recent data suggest breastmilk as a ‘flavour bridge’, whereby repeated exposures to novel flavours from breastmilk in early life could increase children’s acceptance to new foods and shape their future taste preferences [33]. The value of breastfeeding is increasingly recognised and, in recent years, the Baby Friendly Hospital Initiative (BFHI) has been started to protect, promote and support breastfeeding. This includes the provision of training to all maternity ward staff to provide breastfeeding counselling, consultation and support to mothers in local hospitals [34]. Additionally, there is a move encouraging corporate policies towards having a flexible scheduling and provision of designated private spaces for mothers to breastfeed or express breastmilk at workplaces [35].

## 5. Conclusions

In this prospective cohort study, we characterized the complementary foods consumed by GUSTO infants at 6, 9 and 12 months of age by identifying the top food sources of energy and macronutrient and differences in these intakes based on the type of milk feed (BF, MF or FF). The top three food sources of energy of infants from 6 to 12 months of age were infant formula, breastmilk and infant cereals. Differences in nutrient intakes and key food sources of energy and macronutrient could be observed among infants of the three milk-feed types. FF infants were found to consume significantly higher protein (TE%), lower total fat (TE%) and a less varied diet relative to BF and MF infants in general.

An understanding of key food sources of energy and macronutrient provided during complementary feeding may inform local dietary recommendations and policies. From our work, we propose that current recommendations should include an emphasis on introducing a variety of complementary foods from 6 to 12 months of age, independent of the type of milk feeds the infants are on. Additionally, current recommendations could provide more information on healthier snack options, so as to decrease the reliance on nutrient-poor packaged foods as snacks. In future, studies could be extended to assess diets at later time points and explore the implications of early macronutrient intakes on later growth outcomes.

## Figures and Tables

**Table 1:** Comparison of maternal and infant characteristics of single birth infants with at least 1 dietary record at 6, 9 or 12 months of age and those without any.

Characteristics	Total Study Population	Completed at Least a Single-Day Dietary Record at 6, 9 and/or 12 Months	Did Not Complete Any Single-Day Dietary Record at 6, 9 and 12 Months	*p*-Value *
	n (%) ^1^	n (%) ^1^	n (%) ^1^	
**Infant**				
**Gender ^2^**				0.703
Male	619 (52.9)	545 (52.7)	74 (54.8)	
Female	551 (47.1)	490 (47.3)	61 (45.2)	
**Gestational Age ^2^**				**0.006**
<37 weeks	91 (7.8)	72 (7.0)	19 (14.1)	
≥37 weeks	1079 (92.2)	963 (93.0)	116 (85.9)	
**Parity ^2^**				0.235
First child	534 (46.0)	465 (45.3)	69 (51.1)	
Not the first child	628 (54.0)	562 (54.7)	66 (48.9)	
**Duration of any breastfeeding ^2^**				**0.005**
Never	50 (4.7)	48 (4.7)	2 (4.8)	
<3 months	423 (39.6)	396 (38.6)	27 (64.3)	
≥3 months	594 (55.7)	581 (56.7)	13 (31.0)	
**Age of introduction of first foods ^2^**				
≤15 weeks	20 (2.4)	20 (2.4)	Missing data	NA
16–23 weeks	291 (34.6)	291 (34.6)		
24–31 weeks	495 (58.9)	495 (58.9)		
≥32 weeks	34 (4.0)	34 (4.0)		
**Maternal**				
**Maternal age**				**<0.001**
18 to 29	527 (42.6)	419 (40.5)	108 (53.5)	
30 to 34	416 (33.6)	352 (34.0)	64 (31.7)	
>34	294 (23.8)	264 (25.5)	30 (14.9)	
**Ethnicity**				0.012
Chinese	691 (55.9)	594 (57.4)	97 (48.0)	
Malay	322 (26.0)	253 (24.4)	69 (34.2)	
Indian	224 (18.1)	188 (18.2)	36 (17.8)	
**Education ^2^**				**<0.001**
None/Primary/Secondary	381 (31.2)	299 (29.2)	82 (41.6)	
Post-secondary	431 (35.3)	360 (35.2)	71 (36.0)	
University and beyond	408 (33.4)	364 (35.6)	44 (22.3)	
**Employment status ^2^**				0.289
Unemployed	358 (29.6)	293 (28.9)	65 (33.0)	
Employed	852 (70.4)	720 (71.1)	132 (67.0)	
**Monthly household income ^2^**				**<0.001**
≤$1999	181 (15.7)	140 (14.5)	41 (22.2)	
$2000–$5999	640 (55.6)	532 (55.0)	108 (58.4)	
≥$6000	331 (28.7)	295 (30.5)	36 (19.5)	
**Body mass index (BMI) at 26 weeks of gestation ^2^**				0.508
<18.5 to 24.9	486 (46.6)	422 (46.2)	64 (48.9)	
25 to 29.9	364 (34.9)	319 (34.9)	45 (34.4)	
>30	194 (18.6)	172 (18.8)	22 (16.8)	
**Alcohol consumption before pregnancy ^2^**				0.677
Yes	410 (35.1)	356 (34.8)	54 (37.0)	
No	758 (64.9)	666 (65.2)	92 (63.0)	
**Alcohol consumption during pregnancy ^2^**				0.986
Yes	20 (1.7)	18 (1.8)	2 (1.4)	
No	1128 (98.3)	985 (98.2)	143 (98.6)	
**Smoking before pregnancy ^2^**				**0.019**
Yes	156 (13.3)	127 (12.4)	29 (19.9)	
No	1014 (86.7)	897 (87.6)	117 (80.1)	
**Smoking during pregnancy ^2^**				0.285
Yes	29 (1.7)	23 (2.2)	6 (4.1)	
No	1140 (97.5)	1000 (97.8)	140 (95.9)	

^1^ reflects count and percentages within column (in brackets) of categorical variables ^2^ Number of missing data for total study population: Infant (n = 1176): n = 6 for ‘Gender’, n = 6 for ‘Gestational Age’, n = 14 for ‘Parity’, n = 109 for ‘Duration of any breastfeeding’, n = 336 for ‘Age of introduction of first foods’, there were no information on the age of introduction of first foods for those who did not provide any dietary data; Maternal (n = 1237): n = 17 for ‘Education’, n = 27 for ‘Employment status’, n = 85 for ‘Monthly household income; ≤$1999 refers to low income range; $2000–$5999 refers to median income range; ≥$6000,refers to high income range, n = 193 for ‘BMI at 26 weeks of gestation’, n = 69 for ‘Alcohol consumption before pregnancy’, n = 89 for ‘Alcohol consumption during pregnancy’, n = 67 for ‘Smoking before pregnancy’, and n = 68 for ‘Smoking during pregnancy’. * *p* values were obtained from chi-squared tests for categorical variables. Statistically significant *p* values < 0.05 were highlighted in bold.

**Table 2:** Cross-sectional analyses of energy and energy-adjusted macronutrient and dietary fibre intakes of all infants, and stratified by type of milk feed.

Dietary Intakes of Energy, Macronutrient and Fibre	Recommended Intakes		All		Breast-Fed (BF)		Mixed-Fed (MF)		Formula-Fed (FF)		*p*-Value (by Milk-Feed Type)
			(n = 760, 893, 907) ^‡^		(n = 120, 94, 57) ^‡^		(n = 160, 149, 125) ^‡^		(n = 480, 650, 725) ^‡^		
			Mean	Standard Deviation (SD)	Mean	SD	Mean	SD	Mean	SD	
**Energy (TE) (kcal)**	EAR of Male/Female (HPB) ^†^										
6 months	600/560		640	158	652	112	647	98	634	182	0.450
9 months	670/620		675	173	580 ^a^	122	683 ^b^	137	687 ^b^	183	**<0.001**
12 months	740/640		761	208	716	187	795	164	759	216	0.050
**Protein (TE%)**	AMDR (IOM) ^†^										
6 months	-		9.9	2.5	7.1 ^a^	0.7	9.2 ^b^	1.8	10.8 ^c^	2.4	**<0.001**
9 months	-		13.1	3.1	10.5 ^a^	3.0	11.6 ^b^	2.8	13.8 ^c^	2.8	**<0.001**
12 months	5–20		14.5	3.4	10.9 ^a^	3.4	12.5 ^b^	2.7	15.1 ^c^	3.2	**<0.001**
**Total Fat (TE%)**	AMDR (IOM) ^†^										
6 months	-		42.0	6.3	49.7 ^a^	3.1	44.4 ^b^	4.3	39.2 ^c^	5.5	**<0.001**
9 months	-		36.9	5.7	38.5 ^a^	5.1	41.7 ^b^	5.6	35.6 ^c^	5.1	**<0.001**
12 months	30–40		34.6	7.5	42.1 ^a^	9.0	41.5 ^a^	7.0	32.8 ^b^	6.3	**<0.001**
**Carbohydrate (TE%)**	AMDR (IOM) ^†^										
6 months	-		48.1	5.3	43.1 ^a^	2.8	46.4 ^b^	3.7	50.0 ^c^	5.2	**<0.001**
9 months	-		50.0	5.7	51.0 ^a^	5.3	46.7 ^b^	5.3	50.6 ^a^	5.5	**<0.001**
12 months	45–65		50.9	7.6	47.0 ^a^	8.1	46.0 ^a^	6.5	52.1 ^b^	7.4	**<0.001**
**Dietary Fibre (g per 1000 kcal of TE) ^§^**											
6 months	-	2.5		2.7	1.1 ^a^	1.6	1.9 ^a^	2.2	3.0 ^b^	2.9	**<0.001**
9 months	-	4.0		3.1	3.9	3.5	4.0	2.9	4.0	3.1	0.981
12 months	-	4.4		3.3	4.7	2.7	4.3	3.8	4.4	3.2	0.743

^†^ EAR (HPB) refers to the estimated average requirement for boys and girls respectively in kcal/day recommended by the Singapore Health Promotion Board (HPB), AMDR (IOM) refers to the acceptable macronutrient distribution range (AMDR) recommended by the Institute of Medicine (IOM) for children aged 1 to 3 years, ‘-’ refers to unavailable data. ^‡^ Refers to number of infants at 6, 9 and 12 months respectively. ^abc^ All mean values within each row with unlike alphabets were significantly different (*p* < 0.05) based on Bonferroni corrected values. Statistically significant *p* values < 0.05 were in bold. ^§^ n numbers for fibre are all (n = 681, 884, 901), breast-fed (n = 97, 94, 57), formula-fed (n = 444, 643, 719), mixed-fed (n = 140, 147, 125) at 6, 9 and 12 months respectively. Smaller n numbers for dietary fibre were recorded as some infants consuming only formula milk, with no other foods during the three time points. The formula milk consumed by these infants does not have the value of dietary fibre stated on the nutritional information panel. Certain brands of infant formula consumed contains dietary fibre in the form of fructo-oligosaccharides (FOS) or galacto-oligosacchraides (GOS).

**Table 3:** Cross sectional analyses of energy, by gender, of all infants and stratified by type of milk feed.

Dietary Intake of Energy (kcal)	Recommended Intakes		All		Breast-Fed		Mixed-Fed		Formula-Fed	
			(n = 760, 893, 907) *		(n = 120, 94, 57) *		(n = 160, 149, 125) *		(n = 480, 650, 725) *	
	EAR (HPB) ^†^		Mean (Standard Deviation)							
	Male	Female	Male	Female	Male	Female	Male	Female	Male	Female
6 months	*600*	*560*	655 (164)	623 (151)	662 (129)	642 (94)	649 (103)	644 (94)	655 (186)	611 (178)
9 months	*670*	*620*	692 (171)	658 (175)	**592 (111)**	**567 (134)**	682 (127)	685 (148)	709 (182)	665 (182)
12 months	*740*	*640*	781 (219)	741 (195)	**720 (129)**	711 (245)	829 (187)	757 (127)	778 (229)	741 (201)
**Dietary Intake of** **Energy (kcal)**	**EAR (HPB) ^†^**		**Percentage of Infants Meeting the EAR (HPB) (%) ^#^**							
	**Male**	**Female**	**Male**	**Female**	**Male**	**Female**	**Male**	**Female**	**Male**	**Female**
6 months	*600*	*560*	62.6	69.5	70.2	90.5	72.1	90.3	57.8	56.1
9 months	*670*	*620*	50.2	56.2	**18.4**	**33.3**	44.9	67.2	56.2	57.1
12 months	*740*	*640*	54.3	67.3	**46.9**	52.0	71.2	84.2	51.9	65.6

* Refers to number of infants at 6, 9 and 12 months respectively. ^†^ Estimated energy requirement (EAR) of the local Health Promotion Board (HPB, in italics). All mean energy intakes are above the EAR for 6, 9 and 12 months, except for 9-month-old breast-fed infants and 12-month-old male breast-fed infants (numbers in bold). Similarly, a much lower percentage of the same group of infants were observed to meet the EAR (numbers in bold). ^#^ Percentage of infants not meeting the EAR can be calculated by subtracting the reported number from 100%. For instance, the percentage of male breast-fed infants not meeting the EAR, at 6 months = 100% − 70.2% = 29.8%.

## Supplementary Materials

The following are available online at http://www.mdpi.com/1660-4601/15/3/488/s1, Figure S1: Flowchart of participants included in cross-sectional analyses; Figure S2: Prevalence of various milk feed groups at each time point; Figure S3: Cross sectional analyses of top food sources of dietary fibre of GUSTO infants at 6, 9 and 12 months of age; Table S1: Food group classifications of infants from 6–12 months of the GUSTO study; Table S2: Sources of energy and macronutrient among all infants at 6, 9 and 12 months of age of the GUSTO study; Table S3: Sources of energy and macronutrient among breast-fed, formula-fed and mixed-fed infants at 6 months of age of the GUSTO study; Table S4: Sources of energy and macronutrient among breast-fed, formula-fed and mixed-fed infants at 9 months of age of the GUSTO study; Table S5: Sources of energy and macronutrient among breast-fed, formula-fed and mixed-fed infants at 12 months of age of the GUSTO study.
