# Supplementary material for: Food Sources of Energy and Macronutrient Intakes among Infants from 6 to 12 Months of Age: The Growing Up in Singapore Towards Healthy Outcomes (GUSTO) Study
Source: Int J Environ Res Public Health. 2018 Mar 10;15(3):488. doi: 10.3390/ijerph15030488 (PMC5877033; doi:10.3390/ijerph15030488)
Supplement: Supplementary file 1 [file ijerph-15-00488-s001.docx]

**Table S1.** Food-group classifications of infants from 6–12 months from the GUSTO study.

| Breastmilk | Fruits and juices | Oils and fats |
| --- | --- | --- |
| Breastmilk | Fresh fruits | Blended oil |
| **Beverages** | Fresh juices ^p^ | MUFA-/PUFA-based oils |
| Sweetened drinks ^a^ | Dried, preserved fruits and others ^q^ | Butter/Ghee |
| Soyabean drink | **Infant products ^r^** | Margarine, peanut butter |
| Traditional drinks ^b^ | Infant cereals | Others (salad dressing, mayo) |
| Water | Infant formula milks | **Soups and gravies** |
| Other type of commonly consumed drinks ^c^ | Infant biscuits | Cream/condensed/powder type |
| **Breads** | Infant bottled/canned desserts | Clear soup |
| White bread | Infant bottled/canned fruits | Gravy, curry based |
| Wholemeal bread | Infant bottled/canned meats/vegetables | Gravy, soya sauce based |
| Bread with fillings/toppings ^d^ | **Legumes and pulses** | Pasta sauce ^ae^ |
| Other breads including ethnic breads ^e^ | Beancurds ^s^ | Sauces and others ^af^ |
| **Confectionery** | Legumes/lentils | **Rice and grain alternatives** |
| Sugar/syrup/condensed milk | Nuts and seeds ^t^ | White rice, plain |
| Sweet spreads and others ^f^ | **Meats** | Brown rice, plain |
| **Desserts and snacks** | Poultry (boiled and steamed) ^u^ | Flavoured rice ^ag^ |
| Cakes/biscuits/local snacks ^g^ | Poultry (deep-fried) ^v^ | Rice porridge ^ah^ |
| Savoury snacks ^h^ | Pork/beef/lamb (boiled and steamed) ^w^ | Breakfast cereals |
| Sweet snacks ^i^ | Pork/beef/lamb (deep-fried) ^x^ | Oats porridge |
| **Eggs** | Meat products ^y^ | Other grains ^ai^ |
| Eggs ^j^ | Other meats and innards ^z^ | **Vegetables** |
| **Fast foods** | **Milk and Dairy** | Broccoli, cauliflower |
| Burgers | Milk ^aa^ | Green leafy |
| Pizza | Malted drinks | Yellow/orange/red ^aj^ |
| **Fish and seafood** | Yoghurt and cultured drinks | Starchy vegetables/gourds ^ak^ |
| Fish (boiled and steamed) ^k^ | Cheese | Potatoes (deep-fried, fast food) ^al^ |
| **Fish and seafood (Cont.)** | **Noodles and pasta** | **Vegetables (Cont.)** |
| Fish (deep-fried)^l^ | Noodles in soup ^ab^ | Fruit-bearing vegetables and others ^am^ |
| Seafood ^m^ Fried noodles ^ac^ | | |
| Fish and seafood products ^n^ | Pasta ^ad^ |  |
| Dried/preserved fish/seafood products ^o^ |  |  |

a Includes glucolin water, commercial non-infant fruit juices; b Includes chrysanthemum tea, barley water , gripe water; c Includes coffee, tea, rice milk; d Includes raisin bread, pizza bun, cream bun; e Includes chapati, thosai, red bean pow; f Includes hazelnut spread, jam, yeast extracts; g Includes donut, kuih bolu, Swiss roll; h Includes dim sum, fried salty snacks; i Includes ice-cream, puddings, chocolate; j Includes chicken eggs, quail eggs; k Includes steamed or boiled threadfin, salmon; l Includes deep-fried threadfin, pomfret; m Includes prawn, squid, scallop; n Includes crabmeat, fishball; o Includes dried scallop, dried anchovy, canned tuna; p Includes pure fruit juice, commercial baby fruit juices; q Includes raisins, red dates, wolfberry; r Commercial infant products; s Includes vegetarian mock meat, soya bean curd, tofu; t Includes peanuts, tree nuts, sunflower seeds; u Includes boiled, grilled, roasted, stewed, steamed chicken or duck; v Includes deep-fried chicken; w Includes boiled, stir-fried pork, beef or lamb; x Includes deep-fried pork lard; y Includes char siew, beef ball, chicken nuggets; z Includes chicken liver, pork liver, frog leg meat; aa Includes milk from cow and goat sources; ab Includes noodles made from wheat and rice flour such as yellow noodles and vermicelli in soup; ac Includes stir-fried noodles made from wheat and rice flour such as yellow noodles and vermicelli; ad Includes macaroni, spaghetti and commercial infant pasta products; ae Includes tomato and cream-based pasta sauce; af Includes sambal, tamarind; ag Includes chicken rice, briyani rice, sushi rice; ah Includes white, brown rice porridge and instant rice porridge; ai Includes millet, barley, semolina, corn, rice flour; aj Includes carrots, tomatoes and capsicums; ak Includes pumpkins, boiled potatoes and zucchini; al Includes commercially prepared mashed potato, french fries, hash brown; am Includes asparagus, lotus root, white fungus.


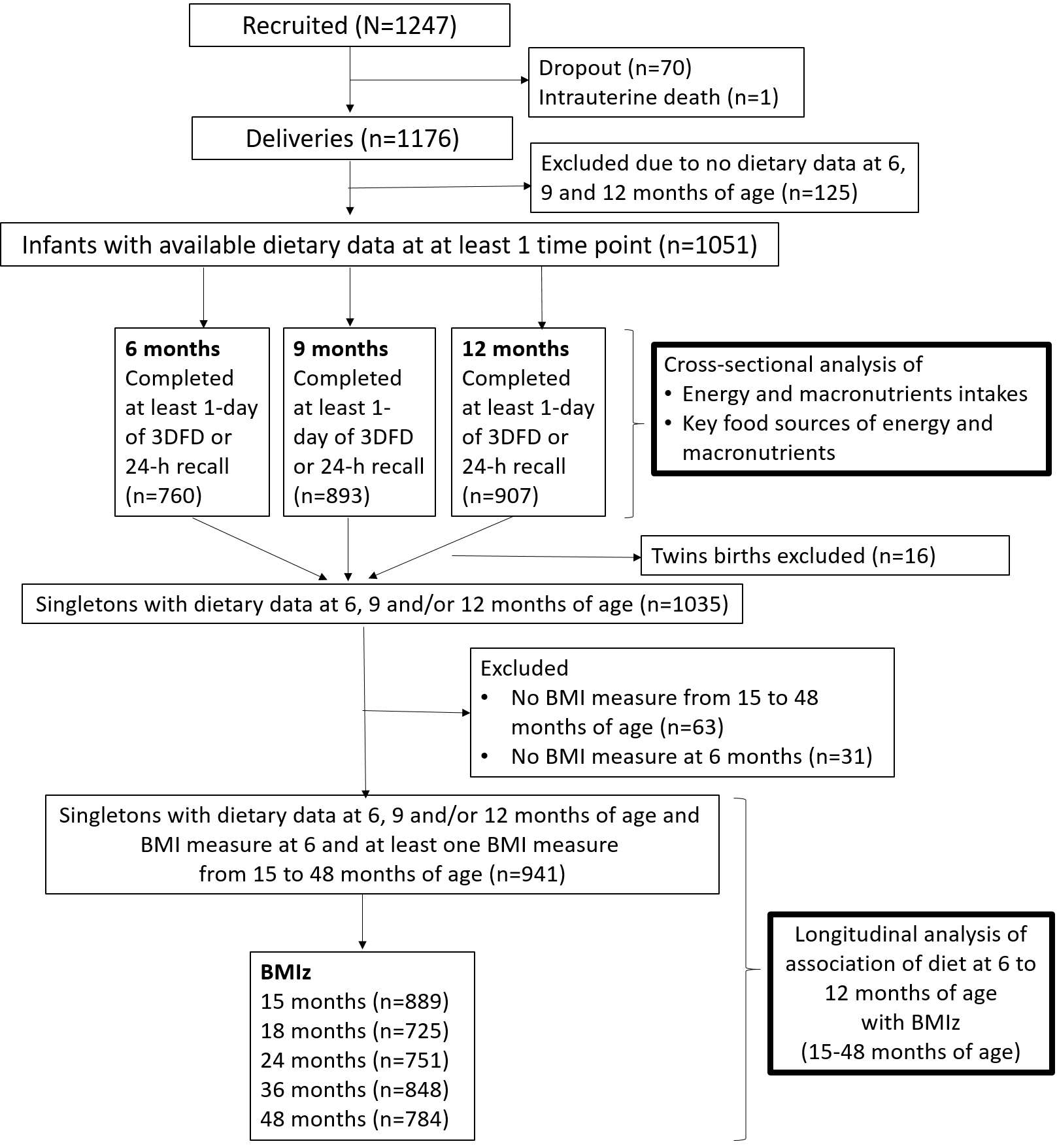


**Figure S1.** Flowchart of participants included in cross-sectional analyses.

**
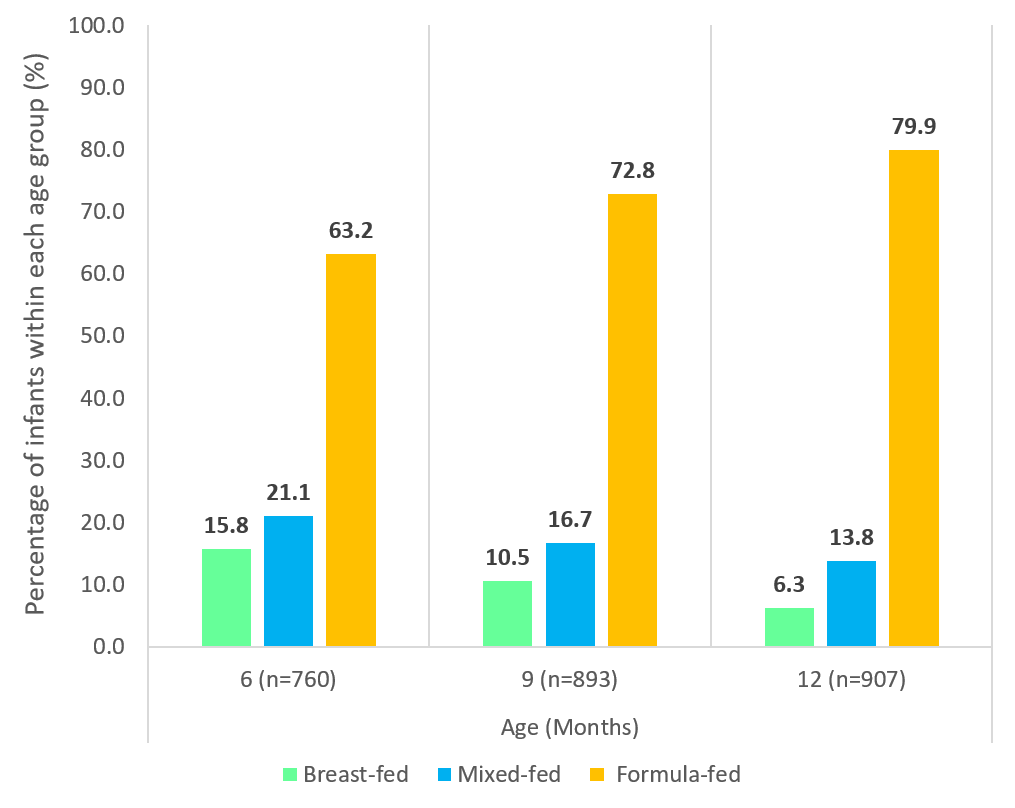
Figure S2.** Prevalence of various milk-feed groups at each time point.

**Table S2.** Sources of energy and macronutrients among all infants at 6, 9 and 12 months of age from the GUSTO study.

| Energy | 6 months (n = 760) | % Contribution | 9 months (n = 893) | % Contribution | 12 months (n = 907) | % Contribution |
| --- | --- | --- | --- | --- | --- | --- |
| **Rank** ^1^ | **Food Group** |  | **Food Group** |  | **Food Group** |  |
| 1 | Infant Formula | 59.9 | Infant Formula | 60.0 | Infant Formula | 57.1 |
| 2 | Breastmilk | 26.4 | Breastmilk | 13.8 | Breastmilk | 9.5 |
| 3 | Infant cereals | 8.0 | Infant cereals | 7.8 | Infant cereals | 5.4 |
| 4 | - |  | Rice porridge | 3.1 | Rice Porridge | 4.0 |
| 5 | - |  | Fish (steamed or boiled) | 1.6 | Fish (steamed or boiled) | 2.0 |
| 6 | - |  | Infant biscuits | 1.5 | Cakes/biscuits/local snacks | 1.9 |
| 7 | - |  | Fresh fruits | 1.4 | Plain white rice | 1.9 |
| 8 | - |  | Cakes/biscuits/local snacks | 1.1 | Pork/beef/lamb (steamed or boiled) | 1.7 |
| 9 | - |  | Pork/beef/lamb (steamed or boiled) | 1.0 | Fresh fruits | 1.6 |
| 10 | - |  | - |  | Eggs | 1.4 |
| 11 | - |  | - |  | Infant biscuits | 1.4 |
| 12 | - |  | - |  | White bread | 1.3 |
| 13 | - |  | - |  | Poultry (steamed or boiled) | 1.1 |
| **Total Fat** | **6 months (n = 760)** | **% Contribution** | **9 months (n = 893)** | **% Contribution** | **12 months (n = 907)** | **% Contribution** |
| **Rank** ^1^ | **Food Group** |  | **Food Group** |  | **Food Group** |  |
|  |  |  |  |  |  |  |
| 1 | Infant Formula | 62.6 | Infant Formula | 66.8 | Infant Formula | 62.4 |
| 2 | Breastmilk | 32.1 | Breastmilk | 19.3 | Breastmilk | 16.0 |
| 3 | Infant cereals | 3.1 | Infant cereals | 3.4 | Eggs | 2.8 |
| **Table S2 Cont.** | | | | | | |
| **Total Fat (Cont.)** | **6 months (n = 760)** | **% Contribution** | **9 months (n = 893)** | **% Contribution** | **12 months (n = 907)** | **% Contribution** |
| **Rank** ^1^ | **Food Group** |  | **Food Group** |  | **Food Group** |  |
| 4 | - |  | Fish (steamed or boiled) | 1.6 | Pork/beef/lamb (steamed or boiled) | 2.7 |
| 5 | - |  | Pork/beef/lamb (steamed or boiled) | 1.6 | Infant cereals | 2.3 |
| 6 | - |  | Eggs | 1.5 | Cakes/biscuits/local snacks | 1.9 |
| 7 | - |  | - |  | Fish (steamed or boiled) | 1.8 |
| 8 | - |  | - |  | Poultry (steamed or boiled) | 1.3 |
| **Protein** | **6 months (n = 760)** | **% Contribution** | **9 months (n = 893)** | **% Contribution** | **12 months (n = 907)** | **% Contribution** |
| **Rank** ^1^ | **Food Group** |  | **Food Group** |  | **Food Group** |  |
| 1 | Infant Formula | 63.0 | Infant Formula | 56.9 | Infant Formula | 50.6 |
| 2 | Breastmilk | 18.8 | Fish (steamed or boiled) | 7.9 | Fish (steamed or boiled) | 9.6 |
| 3 | Infant cereals | 10.3 | Breastmilk | 7.8 | Infant cereals | 4.8 |
| 4 | Fish (steamed or boiled) | 1.6 | Infant cereals | 7.7 | Pork/beef/lamb (steamed or boiled) | 4.8 |
| 5 | - |  | Pork/beef/lamb (steamed or boiled) | 3.4 | Breastmilk | 4.4 |
| 6 | - |  | Rice porridge | 3.0 | Poultry (steamed or boiled) | 3.9 |
| 7 | - |  | Poultry (steamed or boiled) | 1.9 | Rice porridge | 3.5 |
| 8 | - |  | Eggs | 1.6 | Eggs | 3.0 |
| 9 | - |  | - |  | White bread | 1.3 |
| 10 | - |  | - |  | Plain white rice | 1.1 |
| 11 | - |  | - |  | Dried/preserved fish and seafood products | 1.0 |
| **Table S2 Cont.** | | | | | | |
| **Carbohydrates** | **6 months (n = 760)** | **% Contribution** | **9 months (n = 893)** | **% Contribution** | **12 months (n = 907)** | **% Contribution** |
| **Rank** ^1^ | **Food Group** |  | **Food Group** |  | **Food Group** |  |
| 1 | Infant Formula | 56.7 | Infant Formula | 55.9 | Infant Formula | 55.5 |
| 2 | Breastmilk | 22.9 | Breastmilk | 11.3 | Infant cereals | 7.7 |
| 3 | Infant cereals | 11.9 | Infant cereals | 11.2 | Breastmilk | 6.6 |
| 4 | Infant biscuits | 1.2 | Rice porridge | 5.1 | Rice porridge | 6.4 |
| 5 | Rice porridge | 1.0 | Infant biscuits | 2.3 | Plain white rice | 3.3 |
| 6 | Fresh fruits | 1.0 | Fresh fruits | 2.3 | Fresh fruits | 2.7 |
| 7 | - |  | Plain white rice | 1.6 | Cakes/biscuits/local snacks | 2.2 |
| 8 | - |  | Starchy vegetables and gourds | 1.5 | Infant biscuits | 2.0 |
| 9 | - |  | Cakes/biscuits/local snacks | 1.4 | White bread | 2.0 |
| 10 | - |  | - |  | Starchy vegetables and gourds | 1.6 |
| 11 | - |  | - |  | Noodles in soup | 1.0 |
| **Dietary Fibre** | **6 months (n = 760)** | **% Contribution** | **9 months (n = 893)** | **% Contribution** | **12 months (n = 907)** | **% Contribution** |
| **Rank** ^1^ | **Food Group** |  | **Food Group** |  | **Food Group** |  |
| 1 | Infant formula | 48.0 | Infant formula | 27.1 | Infant formula | 17.9 |
| 2 | Infant cereals | 9.6 | Fresh fruits | 12.2 | Fresh fruits | 13.1 |
| 3 | Fresh fruits | 9.3 | Infant cereals | 8.7 | Rice porridge | 9.8 |
| 4 | Yellow/orange/red vegetables | 5.7 | Rice porridge | 8.3 | Legumes and lentils | 9.4 |
| 5 | Starchy vegetables and gourds | 5.0 | Yellow/orange/red vegetables | 7.7 | Yellow/orange/red vegetables | 7.1 |
| **Table S2 Cont.** | | | | | | |
| **Dietary Fibre (Cont.)** | **6 months (n = 760)** | **% Contribution** | **9 months (n = 893)** | **% Contribution** | **12 months (n = 907)** | **% Contribution** |
| **Rank** ^1^ | **Food Group** |  | **Food Group** |  | **Food Group** |  |
| 6 | Infant biscuits | 3.2 | Legumes and lentils | 7.4 | Starchy vegetables and gourds | 5.8 |
| 7 | Legume and Lentils | 3.1 | Starchy vegetables and gourds | 6.3 | Infant cereals | 5.4 |
| 8 | Broccoli and cauliflower | 3.1 | Broccoli and cauliflower | 3.7 | Broccoli and cauliflower | 4.0 |
| 9 | Rice porridge | 2.9 | Infant biscuits | 3.2 | White bread | 3.2 |
| 10 | Infant bottled (canned fruits) | 1.9 | Green leafy vegetables | 1.9 | Green leafy vegetables | 3.0 |
| 11 | Infant bottled (canned meat and vegetables) | 1.3 | Other breads including ethnic breads | 1.4 | Infant biscuits | 2.5 |
| 12 | Plain brown rice | 1.1 | Cakes/biscuits/local snacks | 1.3 | Cakes/biscuits/local snacks | 2.2 |
| 13 | Wholemeal bread | 1.1 | Oats porridge | 1.2 | Other breads including ethnic breads | 1.6 |
| 14 | - |  | - |  | Other grains | 1.5 |
| 15 | - |  | - |  | Noodles in soup | 1.5 |
| 16 | - |  | - |  | Plain white rice | 1.3 |
| 17 | - |  | - |  | Oats porridge | 1.2 |
| 18 | - |  | - |  | Plain brown rice | 1.0 |

^1^ Rank refers to the order of a specific food group based on its contribution (by descending order) to the specific macronutrient/ dietary fibre. For example, infant formula is ranked first among 6-month-old infants’ energy intakes as it contributes the greatest amount of total energy intake (59.9% of total energy).

**
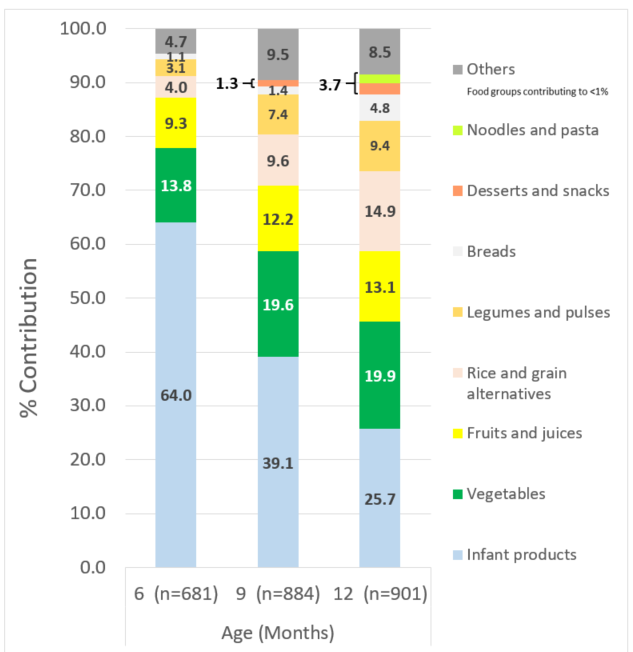
**

**Figure S3.** Cross-sectional analyses of top food sources of dietary fibre of GUSTO infants at 6, 9 and 12 months of age.

**Table S3.** Sources of energy and macronutrients among breast-fed, formula-fed and mixed-fed infants at 6 months of age from the GUSTO study.

| Energy | Breast-fed (n = 120) | % Contribution | Mixed-fed (n = 160) | % Contribution | Formula-fed (n = 480) | % Contribution |
| --- | --- | --- | --- | --- | --- | --- |
| **Rank** ^1^ | **Food Group** |  | **Food Group** |  | **Food Group** |  |
| 1 | Breastmilk | 91.9 | Breastmilk | 55.3 | Infant formula | 84.1 |
| 2 | Infant cereals | 4.5 | Infant formula | 32.9 | Infant cereals | 9.5 |
| 3 | - |  | Infant cereals | 6.2 | Infant biscuits | 1.0 |
| **Total Fat** | **Breast-fed (n = 120)** | **% Contribution** | **Mixed-fed (n = 160)** | **% Contribution** | **Formula-fed (n = 480)** | **% Contribution** |
| **Rank** ^1^ | **Food Group** |  | **Food Group** |  | **Food Group** |  |
| 1 | Breastmilk | 97.5 | Breastmilk | 61.8 | Infant formula | 93.8 |
| 2 | Infant cereals | 1.4 | Infant formula | 32.9 | Infant cereals | 4.0 |
| 3 | - |  | Infant cereals | 2.4 | - |  |
| **Protein** | **Breast-fed (n = 120)** |  | **Mixed-fed (n = 160)** |  | **Formula-fed (n = 480)** |  |
| **Rank** ^1^ | **Food Group** | **% Contribution** | **Food Group** | **% Contribution** | **Food Group** | **% Contribution** |
| 1 | Breastmilk | 86.2 | Breastmilk | 45.3 | Infant formula | 81.0 |
| 2 | Infant cereals | 7.7 | Infant formula | 36.7 | Infant cereals | 11.2 |
| 3 | Fish (steamed or boiled) | 1.1 | Infant cereals | 8.7 | Fish (steamed or boiled) | 1.8 |
| 4 | - |  | Pork/beef/lamb (steamed or boiled) | 1.8 | - |  |
| 5 | - |  | Fish (steamed or boiled) | 1.3 | - |  |
|  |  |  |  |  |  |  |
|  |  |  |  |  |  |  |
|  |  |  |  |  |  |  |
| **Table S3 Cont.** | | | | | | |
| **Carbohydrates** | **Breast-fed (n = 120)** | **% Contribution** | **Mixed-fed (n = 160)** | **% Contribution** | **Formula-fed (n = 480)** | **% Contribution** |
| **Rank** ^1^ | **Food Group** |  | **Food Group** |  | **Food Group** |  |
| 1 | Breastmilk | 86.5 | Breastmilk | 51.3 | Infant formula | 76.9 |
| 2 | Infant cereals | 7.6 | Infant formula | 32.1 | Infant cereals | 13.6 |
| 3 | Fresh fruits | 1.1 | Infant cereals | 9.4 | Infant biscuits | 1.5 |
| 4 | - |  | Fresh fruits | 1.5 | Rice porridge | 1.2 |
| **Dietary Fibre** | **Breast-fed (n= 120)** | **% Contribution** | **Mixed-fed (n = 160)** | **% Contribution** | **Formula-fed (n=480)** | **% Contribution** |
| **Rank** ^1^ | **Food Group** |  | **Food Group** |  | **Food Group** |  |
| 1 | Fresh fruits | 26.4 | Infant formula | 31.2 | Infant formula | 55.7 |
| 2 | Infant cereals | 13.8 | Fresh fruits | 18.0 | Infant cereals | 9.1 |
| 3 | Legumes and lentils | 10.3 | Infant cereals | 10.1 | Fresh fruits | 6.0 |
| 4 | Broccoli and cauliflower | 8.8 | Starchy vegetables and gourds | 9.3 | Yellow/orange/red vegetables | 5.6 |
| 5 | Starchy vegetables and gourds | 7.6 | Yellow/orange/red vegetables | 7.1 | Starchy vegetables and gourds | 3.8 |
| 6 | Infant biscuits | 5.2 | Legumes and lentils | 6.8 | Infant biscuits | 3.4 |
| 7 | Yellow/orange/red vegetables | 4.4 | Broccoli and cauliflower | 3.9 | Rice porridge | 2.9 |
| 8 | Rice porridge | 3.9 | Rice porridge | 2.7 | Broccoli and cauliflower | 2.4 |
| 9 | Plain brown rice | 3.7 | Oats porridge | 1.9 | Infant bottled (canned fruits) | 2.4 |
| 10 | Green leafy vegetables | 2.9 | Infant biscuits | 1.9 | Infant bottled (canned meat and vegetables) | 1.7 |
| 11 | Cakes/biscuits/local snacks | 2.6 | Green leafy vegetables | 1.3 | Legumes and lentils | 1.7 |
| 12 | Oats porridge | 2.4 | Other grains | 1.2 | Wholemeal vbread | 1.4 |
| **Table S3 Cont.** | | | | | | |
| **Dietary Fibre (Cont.)** | **Breast-fed (n = 120)** | **% Contribution** | **Mixed-fed (n = 160)** | **% Contribution** | **Formula-fed (n=480)** | **% Contribution** |
| **Rank** ^1^ | **Food Group** |  | **Food Group** |  | **Food Group** |  |
| 13 | Other grains | 1.7 | - |  | - |  |
| 14 | Herbs and supplements | 1.7 | - |  | - |  |
| 15 | Other breads including ethnic breads | 1.4 | - |  | - |  |
| 16 | Infant bottled (canned fruits) | 1.0 | - |  | - |  |
| ^1^ | | | | | | |

^1^ Rank refers to the order of a specific food group based on its contribution (by descending order) to the specific macronutrient/dietary fibre. For example, breastmilk is ranked first among breast-fed infants’ energy intakes as it contributes the greatest amount of total energy intake (91.9% of total energy).

**Table S4**: Sources of energy and macronutrients among breast-fed, formula-fed and mixed-fed infants at 9 months of age from the GUSTO study.

| Energy | Breast-fed (n = 94) | % Contribution | Mixed-fed (n = 149) | % Contribution | Formula-fed (n = 650) | % Contribution |
| --- | --- | --- | --- | --- | --- | --- |
| **Rank** ^1^ | **Food Group** |  | **Food Group** |  | **Food Group** |  |
| 1 | Breastmilk | 68.5 | Breastmilk | 45.1 | Infant formula | 74.5 |
| 2 | Infant cereals | 8.1 | Infant formula | 28.5 | Infant cereals | 7.9 |
| 3 | Fresh fruits | 3.9 | Infant cereals | 7.3 | Rice porridge | 3.2 |
| 4 | Rice porridge | 2.8 | Rice porridge | 2.9 | Fish (steamed or boiled) | 1.6 |
| 5 | Pork/beef/lamb (steamed or boiled) | 1.8 | Fresh fruits | 2.2 | Infant biscuits | 1.6 |
| 6 | Fish (steamed or boiled) | 1.8 | Fish (steamed or boiled) | 1.6 | Cakes/biscuits/local snacks | 1.1 |
| 7 | Infant biscuits | 1.6 | Infant biscuits | 1.2 | Pork/beef/lamb (steamed or boiled) | 1.0 |
| 8 | Starchy vegetables and gourds | 1.6 | Cakes/biscuits/local snacks | 1.0 | - |  |
| 9 | Plain white rice | 1.0 | - |  | - |  |
|  |  |  |  |  |  |  |
| **Total Fat** | **Breast-fed (n = 94)** | **% Contribution** | **Mixed-fed (n = 149)** | **% Contribution** | **Formula-fed (n = 650)** | **% Contribution** |
| **Rank** ^1^ | **Food Group** |  | **Food Group** |  | **Food Group** |  |
| 1 | Breastmilk | 84.3 | Breastmilk | 60.1 | Infant formula | 85.7 |
| 2 | Infant cereals | 3.5 | Infant formula | 28.1 | Infant cereals | 3.5 |
|  |  |  |  |  |  |  |
| **Table S4 Cont.** | | | | | | |
| **Total Fat (Cont)** | **Breast-fed (n = 94)** | **% Contribution** | **Mixed-fed (n =149)** | **% Contribution** | **Formula-fed (n = 650)** | **% Contribution** |
| **Rank** ^1^ | **Food Group** |  | **Food Group** |  | **Food Group** |  |
| 3 | Pork/beef/lamb (steamed or boiled) | 2.8 | Infant cereals | 2.8 | Fish (steamed or boiled) | 1.8 |
| 4 | Fresh fruits | 2.3 | Fish (steamed or boiled) | 1.2 | Eggs | 1.7 |
| 5 | Eggs | 1.2 | Eggs | 1.2 | Pork/beef/lamb (steamed or boiled) | 1.6 |
| 6 | Fish (steamed or boiled) | 1.2 | Pork/beef/lamb (steamed or boiled) | 1.0 | - |  |
|  |  |  |  |  |  |  |
| **Protein** | **Breast-fed (n = 94)** | **% Contribution** | **Mixed-fed (n =149)** | **% Contribution** | **Formula-fed (n = 650)** | **% Contribution** |
| **Rank** ^1^ | **Food Group** |  | **Food Group** |  | **Food Group** |  |
| 1 | Breastmilk | 48.0 | Infant formula | 30.5 | Infant formula | 67.4 |
| 2 | Fish (steamed or boiled) | 12.2 | Breastmilk | 28.3 | Infant cereals | 7.4 |
| 3 | Infant cereals | 9.9 | Fish (steamed or boiled) | 9.2 | Fish (steamed or boiled) | 7.2 |
| 4 | Pork/beef/lamb (steamed or boiled) | 6.1 | Infant cereals | 8.1 | Pork/beef/lamb (steamed or boiled) | 3.3 |
| 5 | Rice porridge | 3.3 | Rice porridge | 3.2 | Rice porridge | 2.9 |
| 6 | Milk | 2.1 | Pork/beef/lamb (steamed or boiled) | 2.4 | Poultry (steamed or boiled) | 1.9 |
|  | | | | | | |
| **Table S4 Cont.** | | | | | | |
| **Protein (Cont)** | **Breast-fed (n = 94)** | **% Contribution** | **Mixed-fed (n = 149)** | **% Contribution** | **Formula-fed (n = 650)** | **% Contribution** |
| **Rank** ^1^ | **Food Group** |  | **Food Group** |  | **Food Group** |  |
| 7 | Fresh fruits | 1.7 | Poultry (steamed or boiled) | 2.3 | Eggs | 1.6 |
|  |  |  |  |  |  |  |
| 8 | Poultry (steamed or boiled) | 1.5 | Legumes and lentils | 1.7 | - |  |
| 9 | Eggs | 1.5 | Eggs | 1.4 | - |  |
| 10 | Infant biscuits | 1.5 | Dried preserved fish and seafood products | 1.2 | - |  |
| 11 | Starchy vegetables and gourds | 1.2 | Milk | 1.1 | - |  |
| **Carbohydrates** | **Breast-fed (n = 94)** | **% Contribution** | **Mixed-fed (n = 149)** | **% Contribution** | **Formula-fed (n = 650)** | **% Contribution** |
| **Rank** ^1^ | **Food Group** |  | **Food Group** |  | **Food Group** |  |
| 1 | Breastmilk | 61.6 | Breastmilk | 36.3 | Infant formula | 68.5 |
| 2 | Infant cereals | 11.3 | Infant formula | 28.3 | Infant cereals | 11.2 |
| 3 | Fresh fruits | 5.3 | Infant cereals | 11.2 | Rice porridge | 5.1 |
| 4 | Rice porridge | 4.5 | Rice porridge | 5.1 | Infant biscuits | 2.4 |
| 5 | Starchy vegetables and gourds | 2.5 | Fresh fruits | 4.1 | Plain white rice | 1.5 |
| 6 | Infant biscuits | 2.5 | Infant biscuits | 2.0 | Fresh fruits | 1.5 |
| 7 | Plain white rice | 1.8 | Plain white rice | 1.7 | Starchy vegetables and gourds | 1.4 |
| **Table S4 Cont.** | | | | | | |
| **Carbohydrates (Cont.)** | **Breast-fed (n = 94)** | **% Contribution** | **Mixed-fed (n = 149)** | **% Contribution** | **Formula-fed (n = 650)** | **% Contribution** |
| **Rank** ^1^ | **Food Group** |  | **Food Group** |  | **Food Group** |  |
| 8 | Cakes/biscuits/local snacks | 1.3 | Starchy vegetables and gourds | 1.5 | Cakes/biscuits/local snacks | 1.4 |
| 9 | Yellow/orange/red vegetables | 1.2 | Cakes/biscuits/local snacks | 1.4 | - |  |
| 10 | Plain brown rice | 1.2 | Legumes and lentils | 1.2 | - |  |
| 11 | Noodles in soup | 1.0 | Other grains | 1.1 | - |  |
|  |  |  |  |  |  |  |
| **Dietary Fibre** | **Breast-fed (n = 94)** | **% Contribution** | **Mixed-fed (n = 149)** | **% Contribution** | **Formula-fed (n = 650)** | **% Contribution** |
| **Rank** ^1^ | **Food Group** |  | **Food Group** |  | **Food Group** |  |
| 1 | Fresh fruits | 30.1 | Fresh fruits | 17.6 | Infant formula | 35.0 |
| 2 | Yellow/orange/red vegetables | 11.1 | Legumes and lentils | 17.2 | Infant cereals | 8.9 |
| 3 | Starchy vegetables and gourds | 10.5 | Infant formula | 9.5 | Rice porridge | 8.7 |
| 4 | Infant cereals | 8.8 | Yellow/orange/red vegetables | 8.5 | Fresh fruits | 8.4 |
| 5 | Rice porridge | 6.8 | Infant cereals | 8.0 | Yellow/orange/red vegetables | 7.0 |
| 6 | Broccoli and cauliflower | 5.6 | Rice porridge | 7.7 | Starchy vegetables and gourds | 5.7 |
| **Table S4 Cont.** | | | | | | |
| **Dietary Fibre (Cont.)** | **Breast-fed (n = 94)** | **% Contribution** | **Mixed-fed (n = 149)** | **% Contribution** | **Formula-fed (n = 650)** | **% Contribution** |
| **Rank** ^1^ | **Food Group** |  | **Food Group** |  | **Food Group** |  |
| 7 | Legumes and lentils | 4.7 | Starchy vegetables and gourds | 6.5 | Legumes and lentils | 5.5 |
| 8 | Green leafy vegetables | 3.1 | Broccoli and cauliflower | 4.3 | Infant biscuits | 3.5 |
| 9 | Plain brown rice | 2.6 | Other breads including ethnic breads | 2.7 | Broccoli and cauliflower | 3.3 |
| 10 | Infant biscuits | 2.5 | Infant biscuits | 2.5 | Green leafy vegetables | 1.7 |
| 11 | Infant bottled (canned fruits) | 2.0 | Green leafy vegetables | 2.3 | Other breads including ethnic breads | 1.3 |
| 12 | Noodles in soup | 2.0 | Other grains | 2.2 | Cakes/biscuits/local snacks | 1.3 |
| 13 | Oats porridge | 1.8 | Oats porridge | 1.7 | Oats porridge | 1.1 |
|  |  |  |  |  |  |  |
| 14 | Pasta | 1.1 | Sweet snacks | 1.4 | White bread | 1.0 |
| 15 | Cakes/biscuits/local snacks | 1.0 | Cakes/biscuits/local snacks | 1.2 | Infant bottled (canned fruits) | 1.0 |
|  |  |  | **Table S4** Cont. |  |  |  |
| **Dietary Fibre (Cont.)** | **Breast-fed (n = 94)** | **% Contribution** | **Mixed-fed (n = 149)** | **% Contribution** | **Formula-fed (n = 650)** | **% Contribution** |
| **Rank** ^1^ | **Food Group** |  | **Food Group** |  | **Food Group** |  |
| 16 | Wholemeal bread | 1.0 | Plain brown rice | 1.1 | - |  |
| 17 | - |  | Dried preserved fruits and others | 1.0 | - |  |

^1^ Rank refers to the order of a specific food group based on its contribution (by descending order) to the specific macronutrient/dietary fibre. For example, breastmilk is ranked first among breast-fed infants’ energy intakes as it contributes the greatest amount of total energy intake (68.5% of total energy).

**Table S5**: Sources of energy and macronutrients among breast-fed, formula-fed and mixed-fed infants at 12 months of age from the GUSTO study.

| Energy | Breast-fed (n=57) | % Contribution | Mixed-fed (n=125) | % Contribution | Formula-fed (n=725) | % Contribution |
| --- | --- | --- | --- | --- | --- | --- |
| **Rank ^1^** | **Food Group** |  | **Food Group** |  | **Food Group** |  |
| 1 | Breastmilk | 60.0 | Breastmilk | 41.6 | Infant formula | 67.1 |
| 2 | Infant cereals | 4.6 | Infant formula | 25.6 | Infant cereals | 5.4 |
| 3 | Milk | 3.9 | Infant cereals | 6.1 | Rice porridge | 4.2 |
| 4 | Cakes/biscuits/local snacks | 3.9 | Rice porridge | 3.0 | Fish (steamed or boiled) | 2.1 |
| 5 | Rice porridge | 3.5 | Plain white rice | 2.3 | Cakes/biscuits/local snacks | 1.9 |
| 6 | Fresh fruits | 3.1 | Fresh fruits | 2.1 | Plain white rice | 1.8 |
| 7 | Noodles in soup | 2.6 | Fish (steamed or boiled) | 2.1 | Pork/beef/lamb (steamed or boiled) | 1.8 |
| 8 | Plain white rice | 2.1 | Cakes/biscuits/local snacks | 1.5 | Infant biscuits | 1.5 |
| 9 | Fish (steamed or boiled) | 1.6 | Eggs | 1.3 | Eggs | 1.5 |
| 10 | Other breads including ethnic breads | 1.6 | Pork/beef/lamb (steamed or boiled) | 1.3 | White bread | 1.4 |
| 11 | Eggs | 1.2 | White bread | 1.1 | Fresh fruits | 1.3 |
| 12 | Pork/beef/lamb (steamed or boiled) | 1.1 | Other breads including ethnic breads | 1.0 | Poultry (steamed or boiled) | 1.1 |
|  |  |  |  |  |  |  |
|  |  |  | **Table S5**. Cont. |  |  |  |
| **Energy** | **Breast-fed (n=57)** | **% Contribution** | **Mixed-fed (n=125)** | **% Contribution** | **Formula-fed (n=725)** | **% Contribution** |
| **Rank ^1^** | **Food Group** |  | **Food Group** |  | **Food Group** |  |
| 13 | Starchy vegetables and gourds | 1.0 | Legumes and lentils | 1.0 | - |  |
| 14 | - |  | Poultry (steamed or boiled) | 1.0 | - |  |
|  |  |  |  |  |  |  |
| **Total Fat** | **Breast-fed (n=57)** | **% Contribution** | **Mixed-fed (n=125)** | **% Contribution** | **Formula-fed (n=725)** | **% Contribution** |
| **Rank ^1^** | **Food Group** |  | **Food Group** |  | **Food Group** |  |
| 1 | Breastmilk | 80.2 | Breastmilk | 59.9 | Infant formula | 77.1 |
| 2 | Milk | 4.2 | Infant formula | 23.7 | Eggs | 3.1 |
| 3 | Cakes/biscuits/local snacks | 2.5 | Eggs | 2.2 | Pork/beef/lamb (steamed or boiled) | 3.1 |
| 4 | Eggs | 1.7 | Infant cereals | 2.2 | Infant cereals | 2.5 |
| 5 | Pork/beef/lamb (steamed or boiled) | 1.5 | Pork/beef/lamb (steamed or boiled) | 1.9 | Cakes/biscuits/local snacks | 2.0 |
| 6 | Infant cereals | 1.4 | Fish (steamed or boiled) | 1.6 | Fish (steamed or boiled) | 2.0 |
| 7 | Other Breads Including Ethnic Breads | 1.0 | Cakes/biscuits/local snacks | 1.1 | - |  |
|  |  |  | **Table S5.** *Cont.* |  |  |  |
| **Total Fat (Cont)** | **Breast-fed (n=57)** | **% Contribution** | **Mixed-fed (n=125)** | **% Contribution** | **Formula-fed (n=725)** | **% Contribution** |
| **Rank ^1^** | **Food Group** |  | **Food Group** |  | **Food Group** |  |
| 8 | Fish (steamed or boiled) | 1.0 | - |  | - |  |
| 9 | Cheese | 1.0 | - |  | - |  |
|  |  |  |  |  |  |  |
| **Protein** | **Breast-fed (n=57)** | **% Contribution** | **Mixed-fed (n=125)** | **% Contribution** | **Formula-fed (n=725)** | **% Contribution** |
| **Rank ^1^** | **Food Group** |  | **Food Group** |  | **Food Group** |  |
| 1 | Breastmilk | 33.4 | Infant formula | 26.3 | Infant formula | 57.0 |
| 2 | Fish (steamed or boiled) | 11.2 | Breastmilk | 23.5 | Fish (steamed or boiled) | 9.3 |
| 3 | Milk | 8.2 | Fish (steamed or boiled) | 11.2 | Pork/beef/lamb (steamed or boiled) | 5.0 |
| 4 | Infant cereals | 5.1 | Infant cereals | 6.1 | Infant cereals | 4.6 |
| 5 | Rice porridge | 4.2 | Poultry (steamed or boiled) | 4.1 | Poultry (steamed or boiled) | 3.9 |
| 6 | Pork/beef/lamb (steamed or boiled) | 3.8 | Pork/beef /lamb (steamed or boiled) | 3.7 | Rice porridge | 3.6 |
| 7 | Eggs | 3.8 | Rice Porridge | 3.0 | Eggs | 3.0 |
|  |  |  | **Table S5**. *Cont.* |  |  |  |
| **Protein (Cont)** | **Breast-fed (n=57)** | **% Contribution** | **Mixed-fed (n=125)** | **% Contribution** | **Formula-fed (n=725)** | **% Contribution** |
| **Rank ^1^** | **Food Group** |  | **Food Group** |  | **Food Group** |  |
| 8 | Poultry (steamed or boiled) | 3.4 | Eggs | 2.9 | White bread | 1.4 |
| 9 | Seafood | 3.1 | Legumes and lentils | 1.9 | Plain white rice | 1.0 |
| 10 | Noodles in soup | 2.8 | Plain white rice | 1.5 | - |  |
| 11 | Cakes/biscuits/local snacks | 2.4 | Yoghurt and cultured drinks | 1.4 | - |  |
| 12 | Dried/preserved fish and seafood products | 1.7 | White bread | 1.3 | - |  |
| 13 | Plain white rice | 1.6 | Other breads including ethnic breads | 1.2 | - |  |
| 14 | Other breads including ethnic breads | 1.5 | Beancurds | 1.2 | - |  |
| 15 | Cheese | 1.3 | - |  | - |  |
| 16 | Fresh fruits | 1.3 | - |  | - |  |
| 17 | White bread | 1.2 | - |  | - |  |
| 18 | Legumes and lentils | 1.2 | - |  | - |  |
| 19 | Beancurds | 1.1 | - |  | - |  |
|  |  |  | **Table S5**. *Cont.* |  |  |  |
| **Protein (Cont)** | **Breast-fed (n=57)** | **% Contribution** | **Mixed-fed (n=125)** | **% Contribution** | **Formula-fed (n=725)** | **% Contribution** |
| **Rank ^1^** | **Food Group** |  | **Food Group** |  | **Food Group** |  |
| 20 | Other grains | 1.0 | - |  |  |  |
| **Carbo-hydrate** | **Breast-fed (n=57)** | **% Contribution** | **Mixed-fed (n=125)** | **% Contribution** | **Formula-fed (n=725)** | **% Contribution** |
| **Rank ^1^** | **Food Group** |  | **Food Group** |  | **Food Group** |  |
| 1 | Breastmilk | 48.1 | Breastmilk | 30.5 | Infant formula | 63.6 |
| 2 | Infant cereals | 7.2 | Infant formula | 27.1 | Infant cereals | 7.4 |
| 3 | Rice porridge | 5.9 | Infant cereals | 9.6 | Rice porridge | 6.6 |
| 4 | Fresh fruits | 5.6 | Rice porridge | 5.3 | Plain white rice | 3.0 |
| 5 | Cakes/biscuits/local snacks | 5.5 | Plain white rice | 4.5 | Fresh fruits | 2.3 |
| 6 | Noodles in soup | 4.8 | Fresh fruits | 3.8 | Infant biscuits | 2.2 |
| 7 | Plain white rice | 4.0 | Cakes/biscuits/local snacks | 2.0 | White bread | 2.1 |
| 8 | Milk | 2.6 | White bread | 1.9 | Cakes/biscuits/local snacks | 2.1 |
|  |  |  | **Table S5**. *Cont.* |  |  |  |
| **Carbo-hydrate (Cont)** | **Breast-fed (n=57)** | **% Contribution** | **Mixed-fed (n=125)** | **% Contribution** | **Formula-fed (n=725)** | **% Contribution** |
| **Rank ^1^** | **Food Group** |  | **Food Group** |  | **Food Group** |  |
| 9 | Other breads including ethnic breads | 2.2 | Other breads including ethnic breads | 1.6 | Starchy vegetables and gourds | 1.6 |
| 10 | Starchy vegetables and gourds | 1.8 | Legumes and lentils | 1.5 | - |  |
| 11 | Other grains | 1.7 | Starchy vegetables and gourds | 1.5 | - |  |
| 12 | Infant biscuits | 1.6 | Infant biscuits | 1.3 | - |  |
| 13 | White bread | 1.6 | Yellow/orange/red vegetables | 1.1 | - |  |
| 14 | Yellow/orange/red vegetables | 1.2 | Noodles in soup | 1.0 | - |  |
| 15 | - |  | Pasta | 1.0 | - |  |
|  |  |  |  |  |  |  |
| **Dietary Fibre** | **Breast-fed (n=57)** | **% Contribution** | **Mixed-fed (n=125)** | **% Contribution** | **Formula-fed (n=725)** | **% Contribution** |
| **Rank ^1^** | **Food Group** |  | **Food Group** |  | **Food Group** |  |
| 1 | Fresh fruits | 21.3 | Legume and Lentils | 18.9 | Infant Formula | 21.7 |
|  |  |  | **Table S5**. *Cont.* |  |  |  |
| **Dietary Fibre (Cont)** | **Breast-fed (n=57)** | **% Contribution** | **Mixed-fed (n=125)** | **% Contribution** | **Formula-fed (n=725)** | **% Contribution** |
| **Rank ^1^** | **Food Group** |  | **Food Group** |  | **Food Group** |  |
| 2 | Legumes and lentils | 10.9 | Fresh fruits | 15.7 | Fresh fruits | 11.9 |
| 3 | Yellow/orange/red vegetables | 8.7 | Yellow/orange/red vegetables | 8.0 | Rice porridge | 10.6 |
| 4 | Noodles in soup | 7.6 | Rice porridge | 6.8 | Legumes and lentils | 7.4 |
| 5 | Rice porridge | 7.2 | Infant cereals | 6.3 | Yellow/orange/red vegetables | 6.8 |
| 6 | Starchy vegetables and gourds | 5.4 | Starchy vegetables and gourds | 6.2 | Starchy vegetables and gourds | 5.7 |
| 7 | Infant cereals | 5.0 | Infant formula | 6.0 | Infant cereals | 5.3 |
| 8 | Other breads including ethnic breads | 4.7 | Broccoli and cauliflower | 5.0 | Broccoli and cauliflower | 3.8 |
| 9 | Broccoli and cauliflower | 4.5 | Other breads including ethnic breads | 3.6 | White bread | 3.5 |
| 10 | Other grains | 4.2 | Green leafy vegetables | 2.8 | Green leafy vegetables | 3.1 |
| 11 | Cakes/biscuits/local snacks | 3.7 | White bread | 2.5 | Infant biscuits | 2.9 |
| 12 | Green leafy vegetables | 2.2 | Other grains | 2.3 | Cakes/biscuits/local snacks | 2.1 |
| 13 | White bread | 2.0 | Pasta | 1.9 | Oats porridge | 1.3 |
|  |  |  | **Table S5**. *Cont.* |  |  |  |
| **Dietary Fibre (Cont)** | **Breast-fed (n=57)** | **% Contribution** | **Mixed-fed (n=125)** | **% Contribution** | **Formula-fed (n=725)** | **% Contribution** |
| **Rank ^1^** | **Food Group** |  | **Food Group** |  | **Food Group** |  |
| 14 | Fruit-bearing vegetables and others | 1.5 | Cakes/biscuits/local snacks | 1.7 | Plain white rice | 1.2 |
| 15 | Infant biscuits | 1.5 | Plain brown rice | 1.5 | Other grains | 1.2 |
| 16 | Plain brown rice | 1.4 | Dried preserved fruits and others | 1.5 | Noodles in soup | 1.1 |
| 17 | Milk | 1.3 | Plain white rice | 1.5 | - |  |
| 18 | Plain white rice | 1.3 | Noodles in soup | 1.1 | - |  |
| 19 | Gravy, curry based | 1.2 | Oats porridge | 1.1 | - |  |
| 20 |  |  | Fruit-bearing vegetables and others | 1.0 | - |  |
|  | | | | | | |

1 Rank refers to the order of a specific food group based on its contribution (by descending order) to the specific macronutrient/dietary fibre. For example, breastmilk is ranked first among breast-fed infants’ energy intakes as it contributes the greatest amount of total energy intake (60% of total energy).
